# Flourishing and integrative emotion regulation: an SDT perspective

**DOI:** 10.3389/fpsyg.2024.1409377

**Published:** 2024-04-25

**Authors:** Randall Curren, Sieun Sienne Park

**Affiliations:** ^1^Department of Philosophy, University of Rochester, Rochester, NY, United States; ^2^Warner Graduate School of Education, University of Rochester, Rochester, NY, United States

**Keywords:** flourishing, emotion, regulation, self-determination theory, integration, basic psychological needs

## Abstract

This paper presents a Self-Determination Theory (SDT) perspective on the relationship between human flourishing and emotion regulation. It argues that SDT’s organismic approach to motivation, development, and wellness enables it to directly address this relationship, placing emotion regulation within comprehensive conceptions of eudaimonic functioning (i.e., flourishing) and regulation (i.e., self-determination). This is in contrast to the dominant goal-directed process model of emotion regulation, which addresses only limited aspects of well-being, ignores forms of motivation that are essential to flourishing, and blurs the line between emotion regulation and other forms of regulation.

## 1 Introduction

In their common uses, the terms *well-being* and *flourishing* pertain to how well a person’s life is going. Lives have multiple aspects that are encompassed by these terms, and the various aspects of well-being or flourishing tend to be functionally interrelated (Bishop, 2015). Competing philosophical theories of well-being have nevertheless been framed as conceptions of what is “ultimately” good for a person (Alexandrova, 2017, pp. 157–161). These theories have revolved around subjective mental states, preference or goal attainment, or things that objectively suit our nature, such as good relationships. Equating well-being with subjective well-being (Diener, 1984) is one of several options in this conflicted theoretical terrain, but for the purposes of researching and promoting well-being we arguably need a more comprehensive conception and measure of it (Ryan and Deci, 2001; Martela and Ryan, 2023). The term *eudaimonic well-being* was first introduced in psychology in 2001 as a comprehensive conception of well-being inspired by Aristotle’s conception of *eudaimonia* or flourishing (Ryan and Deci, 2001), and the term *flourishing* has subsequently come into widespread use. There have been dozens of attempts to define flourishing (Vitterosø, 2016; Martela and Sheldon, 2019), but they coalesce around defining it as follows:

Flourishing = ongoing healthy growth and functioning involving fulfillment of human potential that is in some sense “positive” and is personally meaningful, satisfying, and (at least sometimes) enjoyable.

A widely accepted definition of *emotion regulation* (ER) is that it refers to activities people engage in to influence what emotions they have, when they have them, and how they experience or express them (Gross, 1998, 2015). On its face, this is an over-broad definition. It is over-broad because there are limitless activities in which people might engage to influence the emotions they have, from booking a space flight in order to experience awe to committing suicide to escape unbearable shame, and many of these activities have nothing to do with making oneself less emotionally dysregulated. The concepts of emotional regulation and dysregulation are evidently contrastive, and it is unclear how either could be defined without reference to emotional functioning that is healthy or consistent with well-being or flourishing. A better definition of ER would thus be:

Emotion Regulation = efforts to make emotional functioning healthier or more compatible with well-being or flourishing.

This implies a conceptual relationship between flourishing and emotion regulation, but many questions remain.

If emotions are a category of affective states that are to some extent amenable to regulation (Gross, 2024, pp. 4–5) and flourishing were nothing more than subjective well-being, defined as a preponderance of positive over negative affect, then ER could be a form of affect regulation that contributes directly to flourishing. The goal of ER might be to experience more positive affect, less negative affect, or both, and thereby achieve greater flourishing. Flourishing is not simply subjective well-being, however, so this would be an unacceptably simplistic view of the relationship between flourishing and ER.

Flourishing pertains primarily to qualities of agency and life activities, so understanding the role of ER in flourishing will almost certainly require that ER be understood in the context of agency more broadly. Self-Determination Theory (SDT) offers a helpfully comprehensive framework. Our purpose in what follows is to outline an SDT perspective on *integrative emotion regulation* (IER) and its role in flourishing, contrasting it with the goal-directed process model of ER developed by Gross and colleagues (Gross 1998, 2015; Gross and Ford 2024).

## 2 A self-determination theory perspective on flourishing

SDT is an organismic theory of human motivation, development, and well-being that has been developed over several decades on the basis of widely replicated research (Ryan and Deci, 2017; Ryan, 2023). The explanatory core of SDT’s growing structure of sub-theories is Basic Psychological Needs Theory (BPNT), which posits universal psychological human needs (BPNs) to experience *autonomy* (self-directedness congruent with personal values and sense of self), *relatedness* (a cooperative social climate and affirming relationships), and *competence* (experiencing oneself as capable). Satisfaction of these needs is associated with active fulfillment of agentive, social, and creative potential (Ryan and Deci, 2001; Ryan et al., 2013), and a central, cross-culturally replicated finding in SDT is that the satisfaction of all three BPNs is essential to and predictive of well-being, measured in a variety of ways (Ryan et al., 2023).

Agentive potential and the related need for autonomy are manifested in the innate tendencies of human beings to act, explore, socialize, and self-integrate that SDT refers to as *intrinsically* (i.e., innately and non-instrumentally) motivated. Development of potential is seen as occurring largely through such intrinsically motivated activity in psychologically need-supportive conditions that allow individuals to pursue what interests them, experiencing enjoyment and personal efficacy, while adopting goals and values from their environments through processes of self-integration (Ryan and Deci, 2017; Curren and Ryan, 2020). Intrinsically motivated activity, the internalization and integration it entails, and enactment of integrated values are core characteristics of a flourishing life, as defined above; flourishing involves fulfilling one’s potential in positive ways and SDT explains the inherent meaning, satisfaction, and pleasure in such fulfillment of potential as largely arising though satisfaction of BPNs.

Consider the need for relatedness, which is essentially a need to experience relating to others and others relating to oneself and each other in ways that affirm everyone’s value as persons. Acts of valuing other people for themselves arise from a motivational condition in which such valuing has been integrated into a self that is relatively coherent with respect to cognitive, motivational, and emotional functioning. There is a great deal of evidence indicating that satisfaction of the relatedness need requires such motivation and that instrumentalization of human encounters frustrates the BPN for relatedness (Curren and Ryan, 2020). Instrumental or *extrinsic* life goals (such as wealth, image, and fame) have similarly been shown to yield less self-actualization and vitality, and more depression, anxiety, and physical ill-health than *intrinsic* life goals (such as good relationships, personal growth, and community service) (Ryan and Deci, 2017, pp. 272–292; Bradshaw et al., 2023). Differences in goal orientations are thus predictive of more and less flourishing lives, as are differences in how well-integrated people are. Fuller integration or harmonization of cognitive, motivational, and emotional functioning is only possible when internalized goals and values align with autonomous fulfillment of potential that satisfies all three BPNs. The role of emotion in the integrative functioning characteristic of flourishing gives rise to SDT’s concept of *integrative emotion regulation* (IER).

## 3 Integrative emotion regulation

SDT regards ER as an aspect of the integrative processes through which people form coherent selves, rather than merely strategies to align emotions with goals. As defined in SDT, IER involves taking interest in one’s emotions, tolerating and accepting them, and integrating them with other aspects of a coherent self (Ryan et al., 2006; Ryan and Deci, 2017; Roth et al., 2018). IER is not focused on manipulating, downregulating or reframing emotions, but rather on first understanding their significance for one’s needs, values, and goals. This understanding facilitates greater autonomous regulation and related positive consequences (Schultz and Ryan, 2015; Roth et al., 2019, p. 2). “The combination of freedom to experience emotions as they are and to use emotions as a guide for adaptive behavior is precisely what characterizes emotional integrative functioning,” writes Brenning et al., (2015, p. 573). IER is thus “a way of assimilating emotion-laden experiences” that is facilitated by basic need supports, both developmentally (Brenning et al., 2015) and situationally (Roth et al., 2018; Ryan and Vansteenkiste, 2023, p. 19). SDT research on IER has focused on developmental and situational precursors and its advantages over other types of emotion regulation such as emotional suppression (ES; denying, avoiding, or otherwise pushing away emotions) and emotional distancing (ED; downregulating, reframing, or minimizing emotions) in regulating negative or positive emotions. In short, IER goes beyond a view that emotion should be “managed” so as not to get in the way of one’s goals, treating them instead as important informational inputs to the integrative functioning that is characteristic of flourishing.

## 4 The goal-directed process model of emotion regulation

The more dominant approach to emotion regulation (ER) is essentially a schema for distinguishing types and aspects of regulative strategies and points of entry for them in the unfolding of an emotion (Gross, 1998, 2015; Gross and Ford, 2024). This “process model” relies (Gross, 2024, p. 3) on Moors’ goal-directed theory of emotion (Moors, 2017, 2022), which regards all behavior, including “emotional behavior,” as causally explained by “a dual-process model with a parallel-competitive architecture in which (a) the goal-directed process is the default determinant of non-emotional as well [as] emotional behavior, and (b) the stimulus-driven process is the exception” (Moors, 2022, p. 69). Rather than seeing emotions as stimulus-driven, this “response evaluation theory” sees emotions as arising “during person-situation transactions that have particular meaning to the individual in light of currently active goals” (Gross, 2024, p. 3). Moors and Gross are both explicit (Moors, 2022, p. 65; Gross, 2024, p. 3) in drawing on cybernetic or “control” theories in seeing emotions as “arising through a series of iterative cycles comprising four elements (1) a *situation* (2) *attention* that determines which aspects of the situation are perceived; (3) *evaluation* or *appraisal* of the situation in light of currently active goals; and (4) a *response* to the situation” (pp. 3–4). Negative emotions are seen as arising, like all behaviors, from “the detection of a discrepancy between a stimulus and a goal,” and ER is seen as initiation of “action control cycles” intended to diminish or eliminate the discrepancy (Moors, 2017, p. 72). Building on this, Gross’s *process model of emotion regulation* Gross (1998, 2015) distinguishes four stages of the process and five families of ER strategies distinguished by the stages of emotion generation at which they intervene: situational, attentional (e.g., redirection), cognitive (e.g., reappraisal), and response modulation (e.g., suppression). Adaptive regulation involves actively working on emotions so as to minimize their disruptive influences and maximize their support for goal driven behaviors.

## 5 Discussion

We suggested in our introductory remarks that understanding the role of ER in flourishing will almost certainly require that ER be understood in the context of regulation or self-determination more broadly. We have argued that Self-Determination Theory (SDT) offers a sufficiently comprehensive framework. It situates ER and instrumentally motivated action within a more comprehensive theory of motivation, development, and flourishing that posits key roles in flourishing for intrinsic and integrated forms of motivation that include non-instrumental valuing of persons, relationships, and activities. By contrast, the process model of ER suffers from some critical limitations:

1. The process model treats ER and action generally as instrumental with respect to whatever goals a person has, but, as SDT research has shown, not all goals are equally compatible with flourishing. It follows that successful ER, as the process model understands it, is not necessarily conducive to flourishing and may even suppress it. SDT is able to discriminate *healthy* or *adaptive* ER (i.e., IER) from unhealthy or dysfunctional ER, through its criteria of coherent integrated functioning, but the process model cannot. It is concerned more narrowly with the effectiveness of ER for achieving whatever goal is dominant for a person in a particular situation, regardless of how well the goal is integrated into a coherent self and life.
2. The process model’s reliance on a radically goal-directed theory of emotion implicitly commits it to a wider theory of behavioral causation or *motivation* that ignores well-established findings in motivation science. It is thereby arguably precluded from addressing human flourishing, which necessarily involves people relating to others and engaging in activities in ways that exhibit intrinsic and integrated valuing. By contrast, SDT addresses intrinsically motivated acts and the role of BPNs in motivation, flourishing, and regulating the internalization and integration of goals and values.
3. As noted in the introduction, the process model relies on an over-broad definition of ER, blurring the line between ER and other forms of regulation. In doing so, it implies a wider research agenda and policy reach than is warranted by inviting us to focus on such things as regulating the emotions that students experience in taking tests (Harley and Pekrun, 2024), rather than focusing on providing what students need to flourish, as SDT has (Curren, 2023).
4. Finally, SDT’s comparative studies of IER and other modes of ER suggest that IER has advantages associated with it being more autonomy facilitative, sustainable, and less effortful (Roth et al., 2019). Gross and colleagues could stipulate that individuals can adopt emotional integration as an emotion goal, but their process model lacks the theoretical resources to support this. To meaningfully propose emotional integration as a possible emotion goal within the process model would be to take SDT’s Organismic Integration Theory on board and thereby abandon the radically goal-directed theory of human agency on which the process model is grounded.

## Data availability statement

The original contributions presented in the study are included in the article/supplementary material, further inquiries can be directed to the corresponding author.

## Author contributions

RC: Conceptualization, Writing – original draft, Writing – review & editing. SP: Conceptualization, Writing – review & editing.
